# Physicians’ attitudes toward hypnotics for insomnia: A questionnaire-based study

**DOI:** 10.3389/fpsyt.2023.1071962

**Published:** 2023-02-14

**Authors:** Masahiro Takeshima, Yumi Aoki, Kenya Ie, Eiichi Katsumoto, Eichi Tsuru, Takashi Tsuboi, Ken Inada, Morito Kise, Koichiro Watanabe, Kazuo Mishima, Yoshikazu Takaesu

**Affiliations:** ^1^Department of Neuropsychiatry, Akita University Graduate School of Medicine, Akita, Japan; ^2^Psychiatric and Mental Health Nursing, St. Luke’s International University, Tokyo, Japan; ^3^Department of Neuropsychiatry, Kyorin University School of Medicine, Tokyo, Japan; ^4^Department of General Internal Medicine, St. Marianna University School of Medicine, Kawasaki, Japan; ^5^Katsumoto Mental Clinic, Osaka, Japan; ^6^Department of Neurosurgery, Munakata Suikokai General Hospital, Fukuoka, Japan; ^7^Department of Psychiatry, Kitasato University School of Medicine, Sagamihara, Japan; ^8^Centre for Family Medicine Development, Tokyo, Japan; ^9^Department of Neuropsychiatry, Faculty of Medicine, University of the Ryukyus, Nishihara, Japan

**Keywords:** benzodiazepine, melatonin receptor agonist, non-benzodiazepine, orexin receptor antagonist, questionnaire survey

## Abstract

**Introduction:**

Benzodiazepines and non-benzodiazepines are still widely prescribed despite safety concerns and the introduction of novel hypnotics (orexin receptor antagonists [ORA] and melatonin receptor agonists [MRA]), which may be influenced by physicians’ attitudes toward hypnotics.

**Methods:**

A questionnaire survey was administered to 962 physicians between October 2021 and February 2022, investigating frequently prescribed hypnotics and the reasons for their selection.

**Results:**

ORA were the most frequently prescribed at 84.3%, followed by non-benzodiazepines (75.4%), MRA (57.1%), and benzodiazepines (54.3%). Compared to non-frequent prescribers of hypnotics, a logistic regression analysis showed that frequent ORA prescribers were more concerned with efficacy (odds ratio [OR]: 1.60, 95% confidence interval [CI]: 1.01–2.54, *p* = 0.044) and safety (OR: 4.52, 95% CI: 2.99–6.84, *p* < 0.001), frequent MRA prescribers were more concerned with safety (OR: 2.48, 95% CI: 1.77–3.46, *p* < 0.001), frequent non-benzodiazepine prescribers were more concerned with efficacy (OR: 4.19, 95% CI: 2.91–6.04, *p* < 0.001), and frequent benzodiazepine prescribers were more concerned with efficacy (OR: 4.19, 95% CI: 2.91–6.04, *p* < 0.001) but less concerned with safety (OR: 0.25, 95% CI: 0.16–0.39, *p* < 0.001).

**Discussion:**

This study suggested that physicians believed ORA to be an effective and safe hypnotic and were compelled to prescribe benzodiazepine and non-benzodiazepine frequently, choosing efficacy over safety.

## 1. Introduction

Benzodiazepine (BZ) and non-benzodiazepine (NBZ) increase the risk of dependence with long-term use (1). In recent years, novel hypnotics, such as melatonin receptor agonists (MRA) and orexin receptor antagonists (ORA), with safety profiles have been introduced (2–4), but BZ and NBZ are still commonly prescribed for insomnia in real clinical practice (5, 6). In a study using a large Japanese claims database, 59.5% were reportedly prescribed BZ, and 36.8% were prescribed NBZ as the first hypnotic for insomnia treatment between January 2012 and December 2016 (5).

Several guidelines provide several recommended individual hypnotics for insomnia. Academy of Sleep Medicine (AASM) Clinical Practice guidelines recommended triazolam, zaleplon, and ramelteon for sleep onset insomnia, suvorexant, and doxepin for sleep maintenance insomnia, and temazepam, zolpidem, and eszopiclone for both sleep onset and sleep maintenance insomnia; Clinical Practice Guideline by the American College of Physicians recommended eszopiclone, zolpidem, and suvorexant (7–10). Although there are many types of BZ, BZ recommended in insomnia guidelines are limited to a few drugs, such as triazolam and temazepam, while NBZ, MRA, and ORA seem commonly recommended in many guidelines, despite their small variety (7–10). However, while the AASM Clinical Practice guidelines and Korean Clinical Practice Guideline recommended each hypnotic based on the type of insomnia (7, 9), other guidelines did not clearly show recommended hypnotics according to characteristics of the patients (e.g., the severity of insomnia, physical comorbidity) (8, 10). Further, these guidelines did not provide strategies for when those hypnotics are not effective (7–10).

In this current situation, where the evidence for insomnia treatment is insufficient, physicians’ prescribing behavior for insomnia may be influenced by clinicians’ attitudes, such as preferences toward and beliefs about hypnotics based on their clinical experience. In 2004, National Institute for Clinical Excellence recommended short-acting BZ for insomnia from a cost perspective due to the lack of solid evidence distinguishing between short-acting BZ and NBZ at the time. Yet, NBZ prescriptions increased, and BZ prescriptions decreased in the UK (11, 12). To clarify this, a previous study examined general practitioners’ (GPs) attitudes toward prescribing BZ and NBZ (12). The study showed that GPs believed NBZ was superior to BZ in efficacy and safety. The research team concluded that these GPs’ attitudes might explain the increase in NBZ prescriptions (12). To determine why BZ and NBZ, which have safety concerns, are still commonly prescribed even with the advent of novel hypnotics, it is necessary to investigate recent physicians’ attitudes toward prescribing hypnotics.

To clarify this, we conducted a questionnaire survey to examine recent physicians’ attitudes toward prescribing hypnotics, including MRA and ORA.

## 2. Materials and methods

### 2.1. Study design and participants

This study is an unpaid questionnaire survey of physicians to examine the factors associated with each frequently prescribed class of hypnotic. We sent questionnaires between October 22, 2021 and February 1, 2022, to physicians affiliated with the Japanese Primary Care Association (JPCA) and the All Japan Hospital Association (AJHA) by e-mail, and the Japanese Association of Neuro-Psychiatric Clinics (JAPC) by letter. Members of the JPCA consist of primary care physicians and other healthcare professionals engaged in primary care. Members of the AJHA are representatives of hospitals who have joined the association in agreement with its purpose of contributing to the improvement of public health and the development of local communities by conducting surveys, research, and other activities necessary for the progress and development of hospitals and the fulfillment of their missions. Members of the JAPC are physicians with at least 5 years of clinical experience in psychiatry who manage a clinic with psychiatry as its primary advocacy department or equivalent.

### 2.2. Survey items

The survey items consisted of physician attributes (age groups: 20s, 30s, 40s, 50s, 60s, 70s, and 80s and over), specialty (psychiatric or otherwise), frequently prescribed hypnotics (e.g., BZ and NBZ hypnotics, MRA, and ORA), and reasons for selecting frequently prescribed hypnotics (e.g., effectiveness, appropriate duration of action, safety, familiarity, recommended, and drug price). Questions regarding frequently prescribed hypnotics and the reasons for their use were multiple-choice with no rank order. The questionnaire sent to participants is shown in Supplementary Table 1.

### 2.3. Details of hypnotics

Supplementary Table 2 shows the details of hypnotics that can be prescribed under insurance coverage at the time of the study. Japanese physicians can prescribe all the hypnotics listed in Supplementary Table 2 to patients with insomnia, regardless of whether they are board-certified specialists. BZ was launched between 1967 and 1999, NBZ between 1989 and 2012, MRA between 2010 and 2020, and ORA between 2014 and 2020. Daily drug prices at the maximum dose were roughly less than 50 yen for drugs marketed before 2000 except quazepam, 50–100 yen for drugs marketed between 2000 and 2010, and more than 100 yen for drugs marketed after 2010.

### 2.4. Statistical analysis

Categorical variables are expressed as numbers (%). To examine factors associated with each frequently used drug for insomnia, a binary logistic regression analysis was performed comparing age group, specialty, and reasons for choosing frequently used drugs. *P*-values < 0.05 (two-sided) were considered significant. All statistical analyses were performed with SPSS Statistics 28.0 (IBM Corp., Armonk, NY, USA).

### 2.5. Ethics

The Ethics Committee of St. Luke’s International University (2021-604) approved this study. Informed consent was obtained from the participants in written or electronic form before answering the questionnaire. The study was conducted in accordance with the Declaration of Helsinki.

## 3. Results

In this survey, the response rate from JPCA, AJHA, and JAPC and the overall response rate was 4.73% (251/5,306), 6.62% (168/2,537), 32.1% (543/1,690), and 10.1% (962/9,533), respectively. Table 1 shows the characteristics of the subjects in this study. Most subjects were middle-aged or older, with 29.5% in their 60s, 28.3% in their 50s, and 18.7% in their 40s. On the other hand, a small number of subjects were young adults, with 8.8% in their 30s and 1.2% in their 20s. Among 962 subjects, 26.1% belonged to JPCA, 17.5% to AJHA, and 56.4% to JAPC. For the medical specialty of the subjects, 40.5% specialized in non-psychiatry and 59.5% in psychiatry.

**TABLE 1:** Characteristics of the subjects.

Item	Number (%)
N	962 (100%)
**Age group**	
20s	12 (1.2%)
30s	85 (8.8%)
40s	180 (18.7%)
50s	272 (28.3%)
60s	284 (29.5%)
70s	109 (11.3%)
80s or more	18 (1.9%)
**Affiliated organizations**	
JPCA	251 (26.1%)
AJHA	168 (17.5%)
JAPC	543 (56.4%)
**Specialty**	
Non-psychiatry	390 (40.5%)
Psychiatry	572 (59.5%)

Categorical variables are expressed as numbers (%). AJHA, All Japan Hospital Association; JAPC, Japanese Association of Neuro-Psychiatric Clinics; JPCA, Japanese Primary Care Association.

**FIGURE 1:**
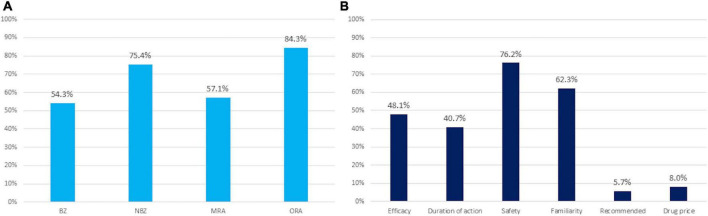
Results of the survey. **(A)** Medications frequently used for insomnia. **(B)** Reasons for selecting medications frequently used for insomnia. Values are expressed as a percent. BZ, benzodiazepine; NBZ, non-benzodiazepine; MRA, melatonin receptor agonist; OR, odds ratio; ORA, orexin receptor antagonist.

Figure 1 shows the results of the survey. Regarding frequently prescribed hypnotics, 84.3% of subjects frequently prescribed ORA, 75.4% of subjects frequently prescribed NBZ, 57.1% of subjects frequently prescribed MRA, and 54.3% of subjects frequently prescribed BZ. Regarding the reason for selecting medications often used for insomnia: 76.2% of subjects answered safety, 62.3% familiarity, 48.1% efficacy, 40.7% appropriate duration of action, 8.0% drug price, and 5.7% recommendation.

Table 2 shows the results of the logistic regression analysis examining factors associated with the frequent prescribing of each hypnotic. Compared to non-frequent BZ prescribers, frequent BZ prescribers were associated with psychiatrist (odds ratio [OR]: 2.67, 95% confidence interval [CI]: 1.83–3.90, <0.001), considering important for efficacy (odds ratio: 4.19, 95% CI: 2.91–6.04, *p* < 0.001), appropriate duration of action (OR: 2.26, 95% CI: 1.56–3.27, *p* < 0.001), familiarity (OR: 3.74, 95% CI: 2.65–5.29, *p* < 0.001), drug price (OR: 3.87, 95% CI: 1.91–7.82), and considering not important for efficacy (OR: 0.25, 95% CI: 0.16–0.39, *p* < 0.001) when selecting medications for insomnia. Compared to non-frequent NBZ prescribers, frequent NBZ prescribers were associated with psychiatrist (OR: 2.43, 95% CI: 1.62–3.67, *p* < 0.001), considered important for efficacy (odds ratio: 4.19, 95% CI: 2.91–6.04, *p* < 0.001), appropriate duration of action (OR: 4.93, 95% CI: 3.11–7.82, *p* < 0.001), and familiarity (OR: 2.29, 95% CI: 1.63–3.22, *p* < 0.001), but not associated with safety, recommended, and drug price when selecting medications for insomnia. Compared to non-frequent MRA prescribers, frequent MRA prescribers were associated with non-psychiatrist (OR: 0.43, 95% CI: 0.31–0.59, *p* < 0.001), considering the appropriate duration of action important (OR: 2.19, 95% CI: 1.58–3.03, *p* < 0.001), safety (OR: 2.48, 95% CI: 1.77–3.46, *p* < 0.001), and familiarity (OR: 1.35, 1.00–1.82, *p* = 0.047), but not associated with age group, efficacy, and recommended when selecting medications for insomnia. Compared to non-frequent ORA prescribers, frequent ORA prescribers were associated with being a psychiatrist (OR: 2.823, 95% CI: 1.83–4.35, *p* < 0.001), considered important for efficacy (OR: 1.602, 95% CI: 1.01–2.54, *p* = 0.044), safety (OR: 4.52, 95% CI: 2.99–6.84, *p* < 0.001), and considering not important for drug price (OR: 0.39, 95% CI: 0.21–0.73, *P* = 0.003) but not associated with age group, efficacy, appropriate duration of action, familiarity, and recommended when selecting medications for insomnia.

**TABLE 2:** Logistic regression analysis examining the factors associated with frequent prescribing of each hypnotic.

	BZ (*N* = 522)		NBZ (*N* = 725)		MRA (*N* = 549)		ORA (*N* = 811)	
	**OR (95% CI)**	***P*** **-value**	**OR (95% CI)**	***P*** **-value**	**OR (95% CI)**	***P*** **-value**	**OR (95% CI)**	***P*** **-value**
**Age group**								
20s	Reference		Reference		Reference		Reference	
30s	8.04 (1.15–56.26)	0.036*	14.37 (2.94–70.23)	<0.001*	0.45 (0.053–3.87)	0.469	0.68 (0.072–6.42)	0.736
40s	9.37 (1.40–62.76)	0.021*	27.03 (5.67–128.82)	<0.001*	0.26 (0.032–2.14)	0.211	0.32 (0.037–2.81)	0.306
50s	9.20 (1.39–61.02)	0.021*	26.12 (5.55–122.82)	<0.001*	0.22 (0.028–1.81)	0.160	0.29 (0.033–2.46)	0.254
60s	11.77 (1.77–78.43)	0.011*	21.65 (4.58–102.4)	<0.001*	0.19 (0.023–1.54)	0.120	0.21 (0.025–1.84)	0.160
70s	27.11 (3.82–192.22)	<0.001*	36.61 (7.09–188.97)	<0.001*	0.22 (0.026–1.81)	0.157	0.20 (0.022–1.80)	0.150
80s and more	9.88 (1.053–92.72)	0.045*	12.55 (1.88–83.89)	0.009*	0.15 (0.014–1.49)	0.104	0.13 (0.012–1.42)	0.094
**Specialty**								
Non-psychiatry	Reference		Reference		Reference		Reference	
Psychiatry	2.67 (1.83–3.90)	<0.001*	1.22 (0.84–1.80)	0.298	0.43 (0.31–0.59)	<0.001*	2.82 (1.83–4.35)	<0.001*
**Reasons for selecting medications frequently used for insomnia**								
**Efficacy**								
No	Reference		Reference		Reference		Reference	
Yes	4.19 (2.91–6.04)	<0.001*	2.43 (1.62–3.67)	<0.001*	0.73 (0.53–1.02)	0.068	1.60 (1.01–2.54)	0.044*
**Appropriate duration of action**								
No	Reference		Reference		Reference		Reference	
Yes	2.26 (1.56–3.27)	<0.001*	4.93 (3.11–7.82)	<0.001*	2.19 (1.58–3.03)	<0.001*	1.135 (0.74–1.75)	0.568
**Safety**								
No	Reference		Reference		Reference		Reference	
Yes	0.25 (0.16–0.39)	<0.001*	0.68 (0.44–1.07)	0.094	2.48 (1.77–3.46)	<0.001*	4.52 (2.99–6.84)	<0.001*
**Familiarity**								
No	Reference		Reference		Reference		Reference	
Yes	3.74 (2.65–5.29)	<0.001*	2.29 (1.63–3.22)	<0.001*	1.35 (1.00–1.82)	0.047*	1.16 (0.77–1.75)	0.476
**Recommended**								
No	Reference		Reference		Reference		Reference	
Yes	0.94 (0.45–1.93)	0.860	2.21 (0.98–4.99)	0.055	1.63 (0.86–3.08)	0.134	1.92 (0.76–4.83)	0.167
**Drug price**								
No	Reference		Reference		Reference		Reference	
Yes	3.87 (1.91–7.82)	<0.001*	1.12 (0.55–2.28)	0.759	0.65 (0.39–1.09)	0.101	0.39 (0.21–0.73)	0.003*

Categorical variables are expressed as numbers (%). *P*-values with significant results (<0.05) are labeled with an asterisk. BZ, benzodiazepine; NBZ, non-benzodiazepine; MRA, melatonin receptor agonist; OR, odds ratio; ORA, orexin receptor antagonist.

## 4. Discussion

This is the first study to examine attitudes toward choice regarding medication for insomnia, including novel hypnotics such as MRA and ORA. The most frequently used medicines for insomnia were ORA, followed by MRA, NBZ, and BZ. Additionally, this study found that frequent ORA prescribers were more concerned with efficacy and safety, frequent MRA prescribers were more concerned with safety, frequent NBZ prescribers were more concerned with effectiveness, and frequent BZ prescribers were more concerned with efficacy but less concerned with safety.

Orexin receptor antagonists was the hypnotic with the highest percentage of frequent prescribers, and frequent ORA prescribers believe ORA is efficacious and safe but expensive. In a study using a large Japanese claims database, 0.4% were prescribed ORA as the first hypnotic drug for insomnia between January 2012 and December 2016 (5). Although this study did not examine prescription frequency by class of hypnotics, this study suggests that ORA prescriptions have expanded rapidly over the past several years in treating insomnia. An American AASM Clinical Practice Guideline weakly recommended suvorexant for sleep maintenance insomnia based on the quality of evidence, the balance of benefits and harms, and patient values and preferences (7). In addition, a recent network meta-analysis (NMA) reported that both suvorexant and lemborexant were significantly superior in efficacy and had no difference in safety compared to placebo and concluded that lemborexant is one of the drugs with a favorable profile (13).

Interestingly, this study was performed before this NMA was published, yet the results were consistent in efficacy and safety. Regarding drug price, this study shows that frequent ORA prescribers were less concerned with drug price. The result is understandable because the highest drug price for hypnotics was that of lemborexant, followed by suvorexant at the time of this study. Although ORA drug prices are indeed high, a previous study conducted in Japan showed that lemborexant was superior to zolpidem in terms of cost-effectiveness (14). Therefore, many physicians may prescribe ORAs frequently because of their efficacy and safety despite the high cost of ORA. This study found that frequent ORA prescribers were likely to be psychiatrists rather than non-psychiatrists. Insomnia is a common comorbidity in patients with psychiatric disorders (10) and a factor that anticipates suicide-related events in patients with psychiatric disorders (15, 16). In addition, patients with psychiatric disorders are associated with long-term use of benzodiazepine receptor agonists (BzRA) (17, 18) and are thus considered a high-risk group for BzRA side effects. For these reasons, psychiatrists look for effectiveness and safety in hypnotics.

Non-benzodiazepine was the hypnotic with the second-highest percentage of frequent prescribers. Frequently, NBZ prescribers believe NBZ is efficacious, has an appropriate duration of action, and is familiar but do not believe it is safe. In a 2005 survey conducted at West Lincolnshire Primary Care Trust in the United Kingdom, which examined GPs’ attitudes toward prescribing BZ and NBZ, GPs believed that NBZ was more effective and safer compared to BZ in treating insomnia (12). However, when the study was conducted, novel hypnotics such as MRAs and ORAs without dependency concerns had not been developed (2–4). A recent NMA reported that eszopiclone, zopiclone, and zolpidem were more effective but had more side effects compared with a placebo in terms of treating insomnia (13). This NMA also reported that although no significant difference was noted in dropout due to adverse events between the eszopiclone and placebo groups, zolpidem and zopiclone had significantly more dropouts due to adverse events than placebo (13). Furthermore, previous studies have reported that NBZ was associated with side effects such as increased risk of falls (19, 20), balance dysfunction (21, 22), and increased risk of road traffic crashes, as noted with BZ (19, 22, 23). In addition, a study in Israel reported that NBZ was associated with an increased risk of long-term use of hypnotics compared with BZ (24). With the advent of novel hypnotics with fewer side effects and with an accumulation of research on the side effects of NBZ, physicians prescribing hypnotics probably no longer believe that NBZ is safe. Frequent NBZ prescribers were more concerned with familiarity than non-frequent NBZ prescribers, probably because NBZ is the second oldest hypnotic after BZ.

Melatonin receptor agonists was the hypnotic with a third of the percentage of frequent prescribers, and frequent MRA prescribers believed MRA to be safe, with an appropriate duration of action, and familiar, but did not believe it efficacious. Given that the safety of MRA has been confirmed by various studies (2, 13, 25), it can be inferred that MRAs are often prescribed by safety-conscious physicians. In fact, this study showed that frequent MRA prescribers were more common among non-psychiatrists, probably because non-psychiatrist insomniacs are more likely to have physical comorbidity than psychiatrist insomniacs. Regarding efficacy, a 2017 meta-analysis reported that ramelteon reduced sleep latency by 9 min compared to placebo (7), but a recent NMA reported that ramelteon did not differ in efficacy from placebo and concluded that ramelteon showed no material benefit for insomnia (13). This lack of robustness of the effect of ramelteon on insomnia may have led to the results of this study. Frequent MRA prescribers believe that MRA has an appropriate duration of action terms of duration of action. Unlike BZ and NBZ, few drugs are classified as MRAs, only ramelteon for insomnia in adults and melatonin for insomnia in children. Nevertheless, one possible reason the duration of action of MRAs was considered adequate may be that ramelteon has no hangover effect (2).

Benzodiazepine was the hypnotic with the lowest percentage of frequent prescribers. Frequently BZ prescribers believe BZ to be unsafe but think it is practical, with an appropriate duration of action, familiarity, and inexpensive. These findings are understandable given that BZ is more effective but less safe than placebo (13), an old and familiar drug, inexpensive drug, and available in various action drugs. Interestingly, approximately half of the physicians prescribe BZ frequently, although they realize the safety issues associated with BZ. Although this is only speculation because this study did not examine pharmacotherapy strategies for insomnia, for patients whose insomnia did not remit with hypnotics other than BZ, BZ may often be changed from or added to the hypnotics. A recent NMA reported that in a head-to-head comparison, short-acting BZ was more effective than lemborexant, suvorexant, and ramelteon in short-term treatment (13).

This study showed that frequent BZ and NBZ prescribers were more concerned with efficacy but not safety. It is not possible to conclude from this survey whether frequent prescribers of these drugs are using them because they do not value safety or whether they were compelled to prescribe them frequently out of necessity, with an understanding of safety issues and an expectation of efficacy. This conclusion is only speculation, but given the repeated warnings about the safety of BZ and NBZ (26), physicians may prescribe BZ and NBZ frequently because insomnia has not improved with other safety hypnotics.

This study has some limitations. First, the survey had a low response rate, especially from JPCA and AJHA, whose members are predominantly non-psychiatrists and were surveyed *via* e-mail. In addition to the low overall response rate, the difference in the response rates between psychiatrists and non-psychiatrists might have affected the results of this study. Second, this study did not examine individual hypnotics frequently used by clinicians. The study results may contain heterogeneity, given that hypnotics classified in the same class may differ in their effects, side effects, and duration of action. Third, because this study used a multiple-choice method of surveying frequently used hypnotics, it was impossible to directly link the factors that the subjects considered important when selecting a hypnotic different from the formula often prescribed. Some subjects may have frequently been prescribed one class of hypnotics for effectiveness and frequently used another for safety. However, the results of this study are generally consistent with the recent NMA regarding efficacy and safety (13), the results regarding familiarity also reflect the timing of the launch of each class of hypnotics in Japan, and the results regarding drug prices reflect the prices of hypnotics at the time of the study, the impact of the lack of a direct link between frequently prescribed hypnotic and the reason for their choice would not be significant. Fourth, the study did not consider comorbidities in patients for whom hypnotics were prescribed. The prescription of one class of hypnotic may be affected by comorbidities. Psychiatrists may avoid prescribing BzRA to patients with schizophrenia, mood disorders, anxiety disorders, and alcohol use disorders since these comorbidities have been reported to be predictors of long-term prescribing of BzRA (17). Further, non-psychiatrists may avoid prescribing suvorexant to patients with antifungals and antivirals or immunocompromised patients because suvorexant is contraindicated in Japan with these comorbidities. Fifth, because this study was conducted on Japanese physicians, caution should be exercised when generalizing the results to physicians in other countries with different healthcare systems or environments. Japan has a universal health insurance system, which allows citizens to easily access medical care and receive treatment with a low financial burden. Therefore, Japanese patients may be more accepting of new, expensive ORA than patients in other countries. Regarding the healthcare environment, Japan was the first country in the world in which ORA was approved, and Japanese physicians may be more familiar with ORA than physicians in other countries. Despite the fact that Japan has a medical system and environment conducive to prescribing ORA, about 3/4 of the physicians prescribed NBZ, and about 1/2 prescribed BZ frequently in this survey. This indicates the limitations in treating insomnia with ORA alone, and these findings may be useful to physicians in countries with different health care systems and environments than Japan. Although the most frequently used medications for insomnia were the newest and most expensive ORA in this study, countries with different healthcare systems or environments may have obtained different results from this study. Sixth, this study lacked data on participants’ actual prescriptions during the study period. Thus, it was not possible to compare participants’ responses with their actual prescriptions.

The study findings suggest that physicians were compelled to prescribe BZ and NBZ frequently for efficacy, disregarding safety. In the future, it is hoped that cognitive-behavioral therapy for insomnia, which has been established to be effective and safe (27, 28), will become more widely used and that evidence will be accumulated regarding treatment strategies for patients who fail to respond to novel hypnotics with a safety profile.

## Data availability statement

The raw data supporting the conclusions of this article will be made available by the authors, without undue reservation.

## Ethics statement

The studies involving human participants were reviewed and approved by the Ethics Committee of St. Luke’s International University (2021-604). The patients/participants provided their written informed consent to participate in this study.

## Author contributions

KIe, KIn, KW, and YT: conceptualization and project administration. YA and YT: methodology. MT: software, resources, and visualization. MT, YA, and YT: formal analysis. YA, KIe, EK, ET, TT, KIn, and MK: investigation. YA, KIe, EK, ET, TT, and MK: data curation. MT and YA: writing—original draft preparation. KIn, KW, and KM: supervision. KM and YT: funding acquisition. All authors read and agreed to the published version of the manuscript.
