# Supplementary material for: Physicians’ attitudes toward hypnotics for insomnia: A questionnaire-based study
Source: Front Psychiatry. 2023 Feb 14;14:1071962. doi: 10.3389/fpsyt.2023.1071962 (PMC9971924; doi:10.3389/fpsyt.2023.1071962)
Supplement: Supplementary file 1 [file Table_1.DOCX]

**Table S1.** English version of the questionnaire

*Q1. Age group (single-choice)*

- 20s
- 30s
- 40s
- 50s
- 60s
- 70s
- 80s or more

*Q2. Specialty (single-choice)*

- Psychiatry
- Other

*Q3. Which class of hypnotics do you prescribe frequently? (multiple-choice)*

- Benzodiazepine
- Non-benzodiazepine
- Melatonin receptor agonist
- Orexin receptor antagonist

*Q4. Reasons for selecting frequently prescribed hypnotics (multiple-choice)*

- Efficacy
- Appropriate duration of action
- Safety
- Familiarity
- Recommended
- Drug price
