# Supplementary material for: Physicians’ attitudes toward hypnotics for insomnia: A questionnaire-based study
Source: Front Psychiatry. 2023 Feb 14;14:1071962. doi: 10.3389/fpsyt.2023.1071962 (PMC9971924; doi:10.3389/fpsyt.2023.1071962)
Supplement: Supplementary file 2 [file Table_2.DOCX]

**Table S2.** Dosage and pricing of available hypnotics

| **Generic name** | **Dose** | **Introduction** | **Drug price^A^** |
| --- | --- | --- | --- |
| Benzodiazepine | | | |
| Brotizolam | 0.25 mg/day | September 1988 | 19.30 yen |
| Estazolam | 1–4 mg/day | December 1975 | 23.40 yen |
| Flunitrazepam | 0.5–2 mg/day^B^ | March 1984 | 12.90 yen |
| Flurazepam | 10–30 mg/day | October 1975 | 9.20 yen |
| Haloxazolam | 5–10 mg/day | January 1981 | 23.90 yen |
| Lormetazepam | 1–2 mg/day | August 1990 | 34.00 yen |
| Nitrazepam | 5–10 mg/day | March 1967 | 14.60 yen |
| Quazepam | 20–30 mg/day | November 1999 | 146.80 yen |
| Rilmazafone | 1–2 mg/day | June 1989 | 23.30 yen |
| Triazolam | 0.125–0.5 mg/day^C^ | April 1983 | 23.40 yen |
|  |  |  |  |
| Non-benzodiazepine | | | |
| Eszopiclone | 1–3 mg/day^D^ | April 2012 | 91.20 yen |
| Zolpidem | 5–10 mg/day | December 2000 | 50.30 yen |
| Zopiclone | 7.5–10 mg/day | June 1989 | 17.00 yen |
|  |  |  |  |
| Melatonin receptor agonist | | | |
| Melatonin^E^ | 1–4 mg/day | June 2020 | 415.60 yen |
| Ramelteon | 8 mg/day | July 2010 | 86.20 yen |
|  |  |  |  |
| Orexin receptor antagonist | | | |
| Lemborexant | 5–10 mg/day | July 2020 | 136.20 yen |
| Suvorexant | 15–20 mg/day^F^ | November 2014 | 109.90 yen |

Note: ^A^Daily drug prices are shown for the maximum dose when the study was conducted. ^B^Elderly patients should be limited to a maximum of 1 mg/day. ^C^Elderly patients should be limited to a maximum of 0.25 mg/day. ^D^Elderly patients should be limited to a maximum of 2 mg/day. ^E^Insurance coverage is available for difficulty falling asleep associated with childhood neurodevelopmental disorders. ^F^Elderly patients should be limited to a maximum of 15 mg/day
